# Pharmacokinetics and toxicity evaluation following oral exposure to bisphenol F

**DOI:** 10.1007/s00204-022-03246-w

**Published:** 2022-04-04

**Authors:** Somin Lee, Kyu Sup An, Hye Jin Kim, Hye Jin Noh, JaeWon Lee, Jiho Lee, Kyung Seuk Song, Chanhee Chae, Hyeon Yeol Ryu

**Affiliations:** 1 Bio Technology Division, GLP 2 Center, Korea Conformity Laboratories (KCL), 8, Gaetbeol-ro 145 beon-gil, Yeonsu-gu, Incheon, 21999 South Korea; 2 Department of Veterinary Pathology, College of Veterinary Medicine, Seoul National University, Seoul, South Korea

**Keywords:** Bisphenol F, Household chemicals, Pharmacokinetics, Repeated exposure toxicity, Genotoxicity

## Abstract

Bisphenol F is a substitute material for bisphenol A and is widely used in household products as a raw material for polycarbonate resin, epoxy resin, and plastic reinforcement. It is known to be mainly used in food containers, thermal paper for receipts, and coatings for water pipes. In some countries, bisphenol F has been detected in drinking water and human urine samples. However, due to the lack of safety evaluation data on bisphenol F, it is difficult to establish appropriate guidelines for the proper use of the substance, and social anxiety is increasing accordingly. This study investigated the use, exposure route, and distribution flow of bisphenol F, a household chemical. To determine the no-observed-adverse-effect level (NOAEL) and target organ of bisphenol F after exposure, a single-dose oral toxicity, dose-range finding (28 day oral), repeated dose toxicity (90 day oral), and genotoxicity (reverse mutation, chromosomal abnormality, in vivo micronucleus test) tests were performed. The pharmacokinetic profile was also obtained. The test results are as follows: in the pharmacokinetic study, it was confirmed that single oral exposure to BPF resulted in systemic exposure; in single oral dose toxicity test, the approximate lethal dose was found to be 4000 mg/kg and confusion and convulsion was shown in the test animals; NOAEL was determined to be 2 mg/kg/day for male and 5 mg/kg/day for female, and the no-observed-effect level (NOEL) was determined to be 2 mg/kg/day for males and 1 mg/kg/day for females, and the target organ was the small intestine; genotoxicity tests confirmed that BPF does not induce genotoxicity.

## Introduction

Bisphenol A (BPA) is a chemical substance with high strength, heat resistance, and transparency (Kang et al. 2006). In the early 1960s, the U.S. Food and Drug Administration (FDA) approved BPA-containing polycarbonates and epoxy resins for use in food storage containers and packaging. Since then, more than 4.26 million tons of BFA has been consumed per year worldwide (as of 2011), with an average annual growth rate of more than 6%. In the Asian market, annual growth is more than 10% (Hartle et al. 2016). Bisphenol A is one of the most used chemicals in the world, with 6.8 million tons of commercial production annually, and it is estimated that more than 1 million tons of bisphenol A are produced and imported annually in Asia and the European Union (Hormann et al. 2014).

Since BPA has been used in the synthesis of polycarbonate plastics, epoxy resins, phenoplast resins, phenolic resins, unsaturated polyester resins, as an antioxidant in thermal paper, polyols, modified polyamides, automobile tires, and flame retardants, it is considered a substance with a high potential for human exposure (Kang et al. 2006; Liao and Kannan 2013; Hormann et al. 2014; Song et al. 2017). The routes of exposure to BFA are as follows: (1) skin and respiratory exposure to producers during the manufacture of related products, (2) inflow from food containers and packaging, (3) ingestion by humans after accumulation in fish due to environmental pollution, and (4) exposure to the environment (this includes the detection of concentrations exceeding the level indicating toxicity to aquatic organisms in leachate from factories and urban waste treatment plants, and wastewater from paper recycling plants and various environments such as dental sealants, indoor and outdoor air, and floor dust) (Tsai 2006).

Since the 1990s, there have been ongoing issues and consequent research on the safety of BPA worldwide. Some scholars have argued that BPA acts as an endocrine disruptor (Staples et al. 1998; Moriyama et al. 2002; Maffini et al. 2006; Heimeier and Shi 2010), and it has been reported to cause decreased fertility and developmental disorders, metabolic disorders, hypertension, and premature sexual maturity (Vandenberg et al. 2007; Nah et al. 2011; Bae et al. 2012; Rochester 2013). In particular, research results have been raised that it has a high sensitivity to infants and young children (Joe and Braun 2011; Mikołajewska et al. 2015; Tewar et al. 2016). Since 2008, the FDA has comprehensively reviewed more than 300 academic studies by expert groups such as Scientific Committees to identify the risk of BPA and strengthen regulations (Board of Scientific Counselors 2008). As regulations on the risk of BPA have been amended, various alternatives have been introduced (Table 1).

**Table 1:**
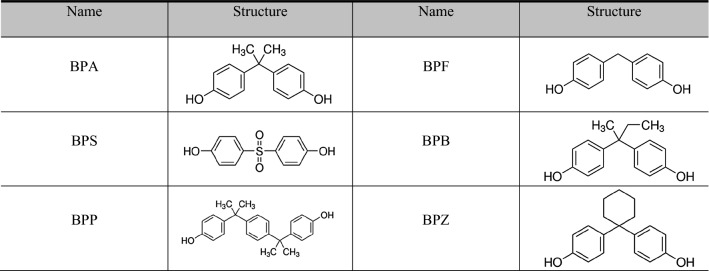
Bisphenol A and its substitutes

Among alternatives, bisphenol F (BPF) is mainly used as an epoxy resin, and is used for food packaging, water pipes, inner coatings of cans, thermal paper receipts, inter alia, and is mainly used as a coating for water supply pipes. In Korea, BPF was detected in higher concentrations than bisphenol A in the water intake of the Han River. In Seoul, the replacement of water pipes using BPF coatings was promoted. Since then, higher BPF levels were detected in the Han River samples than in the Yeongsan River and Nakdong River samples (Yamazaki et al. 2015).

BPF has a structural similarity to BPA, raising questions about the possibility of similarly disturbing the endocrine system (Usman et al. 2019). Table 2 summarizes the ADME, repeated toxicity studies up to 4 weeks, and reproductive risk studies of BPF performed so far. In human exposure studies, bisphenols were detected in human urine worldwide (Chen et al. 2016; Yoo et al. 2016; Yong Eui et al. 2018). The National Health Nutrition Examination survey in the United States 2013–2014 reported BPF in more than 60% of the urine samples, with a geometric mean for all ages of 0.532 μg/l.

Since BPF is mainly used for thermal paper and water pipe coating on receipts, it is continuously exposed to unspecified people regardless of region, occupation, age, or gender.

Despite the lack of safety data on long-term exposure to BPF, BPF is still used for coatings for canned food cans and thermal paper because there is no suitable substitute (Andra et al. 2015; Goldinger et al. 2015). Even though bisphenol substitutes are used in consumer products, the term ‘BPA-free’ is used to convey the perception of ‘eco-friendly’ products. Recently, the relevant risk has been emphasized through scientific studies, and thorough and comprehensive toxicity/safety evaluation of BPF by long-term exposure has become necessary (Eladak et al. 2015).

Globally, the regulation of bisphenol A has been strengthened, while the regulation of alternatives such as BPF is insufficient. BPF are listed as “substances requiring regulation” designated by the International Chemical Office (Chemsec). Therefore, our research team conducted a long-term toxicity study (90-day repeated administration toxicity test), genotoxicity, and pharmacokinetic studies on BPF to resolve concerns and avoidance of chemical products that are prevalent in society.

## Methods

### Preparation of the test substance

The BPF (Lot no.: ZS20190510) was purchased from Orient Chemical Enterprise, China. The test substance displayed properties of crystal foam, with low solubility and dispersibility in polar solvents. After grinding the test material, it was sieved through a 100-mesh screen and processed into powder form. BPF in powder form is uniformly dispersed in corn oil at a 20% concentration; this was therefore, selected as the form and vehicle for the test substance. The test solution showed a change in concentration within ±20%, which is the acceptable range for stability when refrigerated for 7 days, and the coefficient of variation between the upper, middle, and lower layers of the low and high doses was confirmed to be within the homogeneity acceptance range of 5%.

### Pharmacokinetic study

The test was carried out after approval (approval number: IA20-03211) of the Institutional Animal Care and Use Committee (IACUC) of Korean Conformity Laboratories (KCL). BPF was orally administered at a dose of 200 mg/kg body weight (BW), to Sprague–Dawley (SD) rats. To measure the concentration of BPF in the blood, blood was collected at a certain time (0.25, 0.5, 1, 3, 6, 8, 12, 24, 48, and 72 h) after administration, and the concentration of BPF was analyzed using liquid chromatography with tandem mass spectrometry (LC/MS/MS) (LCMS-8060 and Nexera, Shimadzu, Japan). Based on the analysis, changes in blood BPF concentration and pharmacokinetic parameters were calculated via noncompartmental analysis (NCA) (Phoenix WinNonlin^®^, version 6.3, Pharsight Corporation Mountain View, CA, USA).

### Single oral dose toxicity study

The test was carried out after approval (approval number: IA19-01726) of the IACUC of KCL under good laboratory practice (GLP) regulation. BPF was orally administered to SD rats to investigate the toxicity symptoms and the approximate lethal dose. Test solutions were administered once each to male and female rats at doses of 640, 1600, and 4000 mg/kg, and compared with the control group treated with a vehicle control of distilled water. During the experiment, the occurrence of dead animals, general symptoms, and changes in body weight were noted; gross findings of surviving animals were observed at the end of the experiment.

### The 28-day repeated oral dose toxicity study

The test was carried out after approval (approval number: IA19-01920) of the IACUC of KCL and following internationally recognized test guidelines [Organization for Economic Co-operation and Development (OECD 407), 2008] and GLP regulation. To determine the dose of the 90-day repeated oral administration, a toxicity study was performed by repeated oral administration of BPF for 4 weeks. The test group comprised SD male and female rats administered doses of 25.6, 64, 160, 400, and 1000 mg/kg/day, and their results were compared with those of the control group. Body weight changes, feed intake, eye test, urinalysis, clinical pathology (hematologic test, blood biochemical test), organ weight measurement, and macroscopic findings were observed at the time of postmortem.

### The 90-day repeated oral dose toxicity and recovery study

The test was carried out after approval (approval number: IA20-00015) of the IACUC of KCL. In accordance with the OECD testing guidelines (TG408, 2018) and GLP regulation, sub-chronic oral dose toxicity of BPF was studied in male and female SD rats at doses of 2, 10, 50, and 100 mg/kg/day in male and 1, 5, 15, and 30 mg/kg/day in female rats, followed by a 28-day recovery period. All dosing groups were compared with the control group. During the study, the animals were observed for clinical signs (mortalities, daily clinical signs, and weekly detailed clinical observations), functional observations (sensory reactivity to stimuli of different types, assessment of grip strength, and motor activity assessment), weekly body weights, food/water consumption, and then subjected to an ophthalmological examination. At the end of treatment period, the blood and urine were collected for urinalysis, urine sediment analysis, hematology, blood coagulation tests, serum biochemistry, and hormone analysis. Subsequently, the animals were killed and subjected to gross pathological examination, uterus cycle analysis (female only), and organ weight measurement. The organs were preserved for histopathological examination.

### Genotoxicity study

#### Reverse mutation test

To obtain basic data for confirming whether the test substance (BPF) causes carcinogenicity, a microbial reverse mutation test was conducted following internationally recognized test guidelines (OECD 471, 1997) and GLP regulation. In the test, the histidine-requiring strains (TA98, TA100, TA1535, and TA1537) of *Salmonella typhimurium* and WP2uvrA, a tryptophan-requiring strain of *Escherichia coli*, were used. The test substance of the highest concentration was prepared by dissolving the test substance in dimethyl sulfoxide (DMSO), and the test substance of a low concentration was prepared by diluting it step by step in DMSO. Both the direct method and the metabolic activity method were used.

To determine the concentration in the main test, a concentration determination test was conducted in a concentration group of five steps with a mixture of three with 5000 µg/plate as the highest concentration. As a result, growth inhibition was observed in all the strains using the direct method and the metabolic activation method. Compared to the negative control group, no increase in the number of reverse mutated colonies that could be judged as positive was observed. Based on the concentration determination test, the main test was conducted at the following concentrations: TA98 strain of S9 mix (−)/TA1537 of S9 mix (+): 0, 7, 21, 62, 185, and 556 µg/plate.

#### Chromosomal abnormality test

The OECD guidelines 473 (OECD, 2016) informed this test. To evaluate the genotoxicity of BPF, ovarian embryonic cells (CHO-k1 cells) derived from Chinese hamsters were used. The metabolic activation method (+S9 mix) with metabolic activating enzyme system (S9) and the direct method without application (− S9 mix) was used to conduct the chromosomal abnormality test under GLP regulation.

The test substance was dissolved in DMSO to prepare the test substance at the highest concentration and subsequently diluted sequentially for lower concentrations of the test substance. To determine the treatment concentration of the test substance in the main test, a cell proliferation inhibition test at concentrations of 2.29, 6.86, 20.58, 61.73, 185.19, 555.56, 1666.67, and 5000 µg/ml was performed. Three mixtures and concentrations were chosen. The concentrations in the main test were as follows: direct method (− S9 mix; 24 h continuous treatment group): 2.29, 6.86, and 20.58 µg/ml; direct method (− S9 mix; 6 h treatment; 18 h recovery group): 20.58, 61.73, and 185.19 µg/ml; metabolism activity method (+S9 mix; 6 h treatment; 18 h recovery group): 20.58, 61.73, and 185.19 µg/ml.

#### In vivo micronucleus test

To evaluate the genotoxicity of BPF, a micronucleus test was performed using bone marrow cells from Institute of Cancer Research mice following internationally recognized test guidelines (OECD 474, 2016) and GLP regulation. The test was carried out after approval (approval number: IA19-01868) of the IACUC of KCL. The concentration of the test solution was as follows: preliminary test: 2000 mg/kg BW/day, 1000 mg/kg BW/day, 500 mg/kg BW/day, main test: 2000 mg/kg BW/day, 1000 mg/kg BW/day, and 500 mg/kg BW/day. In the preliminary test, three males and three females per group were used. As a result of the preliminary test, symptoms such as abnormal walking were observed in the group of females and males administered the highest concentration of test substance (2000 mg/kg BW/day), but no dead animals were observed. Based on the results of the preliminary test, the main test was conducted with the highest dose concentration of 2000 mg/kg BW/day. In this test, males were used because it was determined that there was no difference in sensitivity to toxicity between males and females.

### Statistical analysis

The differences among the control group and all the dosing groups were analyzed through parametric or non-parametric multiple comparison procedures, as appropriate. Differences were determined to be statistically significant at *p* < 0.05. SPSS for Windows version 12.0 software (SPSS, Chicago, IL, U.S.A.) was used for analysis. Asterisks (*) indicate statistically significant differences compared with the control groups. (1) Analysis of continuous data (body weights, food consumption, water consumption, hematology, blood biochemistry, organ weights): the statistical treatment was conducted assuming normality. The differences among the groups were examined and assumed equal variance using a standard one-way analysis of variance (ANOVA). If the test showed statistical significance, the data were analyzed by parametric multiple comparisons to compare the control group with the experimental groups. If equal variance was obtained, Duncan’s test was used, and if the equal variance was not obtained, Dunnett’s *t*-test was applied. (2) Analysis of non-continuous data (urinalysis): the data were converted by scale conversion and then analyzed using Chi-squared analysis.

## Results

### Pharmacokinetic study

**Fig. 1:**
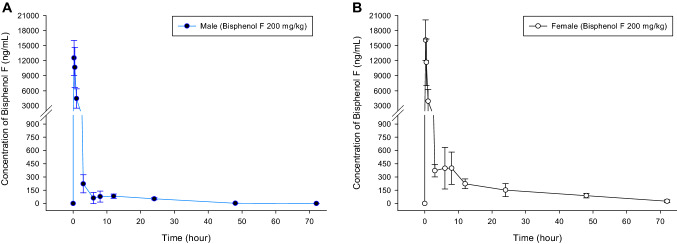
BPF concentration in blood by time points after single-dose administration of 200 mg/kg BPF. **A** male, **B** female. All values are presented as means ± standard errors (*n* = 5 each groups)

The concentration of the BPF increased rapidly 0.25 h after administration, and then decreased rapidly for 3 h thereafter. In males, BPF in blood serum vanished after 72 h, and in females, the drug remained even after 72 h. The concentration of BPF in blood over time is shown in Fig. 1 and the pharmacokinetic parameters are shown in Table 3.

**Table 2:** ADME and toxicological studies on BPF

Study		Contents
ADME (Cabaton et al. 2006)	Absorption/distribution	After a single administration, 46% of the BPF is distributed in the gallbladder within 6 h After 96 h, BPF was distributed in most of the organs and the most distributed in the liver (0.5%) In pregnant rats, BPF is distributed in the uterus, placenta, amniotic fluid, and fetus, and is widely distributed in the lumen of the gastrointestinal tract (8–10%) BPF was efficiently absorbed and distributed to the female genital organs, and the residue passed through the placental barrier at the end of pregnancy
	Metabolism	There are 6 metabolites in urine The major metabolites (over 50%) were in the form of sulfate conjugate
	Excretion	BPF is mainly discharged through urine (43–54%) Rest of BPF is discharged through stool (15–20%)
Toxicity (Higashihara et al. 2007)	28-Day repeated administration oral toxicity test	Weight loss was observed in males 500 mg/kg/females over 20 mg/kg Mild anemia symptoms were observed in females In blood biochemical tests, male and female alkaline phosphatase, gamma-glutamyl transferase increased, total cholesterol decreased, female glucose, albumine, and A/G ratio decreased. A decreasing trend of cholinesterase was observed in male and female An increase in relative weight of liver, kidney, brain, and testis was observed NOAEL was less than 20 mg/kg/day. It is judged to have an effect on the liver
Reproductive toxicity	The effects of BPF and BPS on the endocrine system were confirmed (Ruan et al. 2015; Huang et al. 2016)	
	Bisphenol A substitutes have metabolism, effects, and mechanisms of action, and have estrogen, antiestrogen, androgens, and antiandrogen actions (Bolden 2015)	
	BPF and BPS have adversely affected reproductive function (Ullah et al. 2019; Ijaz et al. 2020)	

As a result of analyzing the difference between males and females, AUC_0-72 h_, C_max_, and Vd value of females were ~ 1.5, 1.3, 1.1 times higher than those of males, respectively. Cl of males was ~ 1.5 times higher than that of females. The elimination half-life was increased in females because females have a higher absorption and lower clearance rate. However, there was no difference in the volume of distribution between both sexes. Unlike other chemicals, BPF has a high vanishing half-life, which is believed to be delayed in excretion from the body. In addition, it is judged that drug loss in females is delayed compared to males because females have a higher absorption rate and a lower excretion rate due to increased elimination half-life. 

### Single oral dose toxicity study

Two males in the 4000 mg/kg group died on the first day of administration. After treatment, symptoms such as salivation and inanimation were observed in the male and female 640 mg/kg group; salivation, inanimation, stupor (male only), and convulsions (female only) were observed in the male and female 640 mg/kg group; and salivation, inanimation, confusion, and convulsions were observed in the male and female 4000 mg/kg group. On the first day of treatment, contamination around the anus was observed in the male 640, 1600, and 4000 mg/kg groups and the female 4000 mg/kg group, and no specific general symptoms were observed thereafter.

**Fig. 2:**
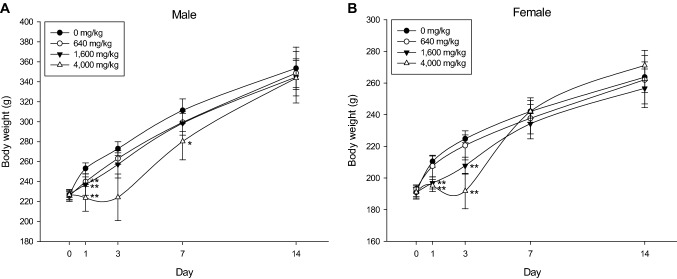
Body weight changes in rats of single oral dose toxicity study. Body weight changes of male (**A**) and female (**B**) rats after single-dose administration of BPF (*n* = 5 per each groups). Error bar represents standard deviation. **Significant changes when compared to its vehicle control group, *p* < 0.01, *significant changes when compared to its vehicle control group, *p* < 0.05

In males, the body weight of the 640, 1600, and 4000 mg/kg groups decreased (*p* < 0.01) on the 1st day after treatment and on the 7th day in the 4000 mg/kg group (*p* < 0.05). There was no difference in body weight on the third day after treatment, but a tendency to lose weight was observed. All animals had recovered to their normal weight by the 14th day. In females, on the 1st and 3rd days after treatment, the body weight of the 1600 and 4000 mg/kg groups decreased (*p* < 0.01), and then recovered normally (Fig. 2). A single oral dose of BFP induced symptoms such as decreased vitality, confusion, convulsions, and weight loss. The approximate lethal dose is considered to exceed 4000 mg/kg in males and 4000 mg/kg in females.

### The 28-day repeated oral dose toxicity study

No dead animals were observed during the experiment, but salivation was observed immediately after administration of the test substance in both male and female groups at > 64 mg/kg/day. Considering the duration and frequency of occurrence, it appears to be an effect of the test substance, but was only observed immediately after administration, and the rats recovered thereafter. This was of no toxicological significance because it was a temporal reaction. Weight loss or a tendency for the body weight to decrease was observed in the male groups receiving > 160 mg/kg/day and the female groups receiving > 64 mg/kg/day. A statistically significant body weight decreases or a weight loss of 10% or more compared to the control group were judged as a toxicological effect of the test substance dosage. As toxicological effects of weight loss were observed in the male > 160 mg/kg/day and female > 64 mg/kg/day dosage groups, the dosages for the 90-day repeated oral dose toxicity study were set to 300, 150, 50, 10, and 2 mg/kg/day for males and 150, 50, 20, 5, and 1 mg/kg/day for females to prevent adverse effects in the rats or target organs upon BPF administration.

### The 90-day repeated oral dose toxicity and recovery study

BPF was administered orally for 90-day repeatedly to determine the observed adverse effects on rats or target organs after BPF administration. Some animals were killed after 90 days of administration (after a recovery period of 4 weeks) to confirm the recovery of the substance.

#### Clinical signs and mortalities

No dead animals were observed during the test. During the administration period, salivation was observed immediately after administration of the test substance in the males dosed > 10 mg/kg/day and females dosed > 15 mg/kg/day. Considering the duration, frequency, and pattern of salivation, it tended to be a dose-dependent effect of the test substance, but it only appeared immediately after administration and not during the recovery period. This is believed to have no toxicological significance as it appears to be due to the temporary promotion of salivation.

#### Detailed clinical observations and functional observations

No significant differences were noted across different groups in both sexes in the main group and the recovery group.

#### Weight measurement

**Fig. 3:**
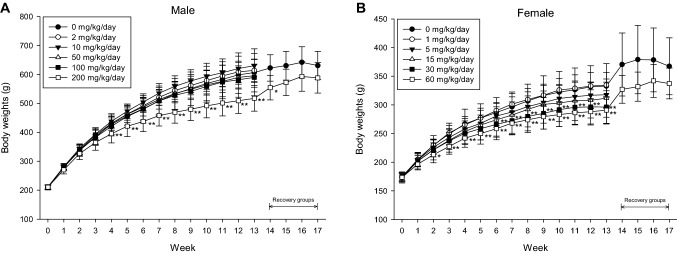
Body weights of rats in 90-day repeated oral toxicity study. Body weight changes of male (**A**) and female (**B**) rats in 13-week repeated oral dose administration of BPF (*n* = 10 per each groups). Error bar represents standard deviation. **Significant changes when compared to its vehicle control group, *p* < 0.01, *significant changes when compared to its vehicle control group, *p* < 0.05

During the administration period, significant weight loss (male 14.7% decrease; female 11–12.6% decrease) was observed in the males receiving > 200 mg/kg/day and in the females receiving > 30 mg/kg/day. During the recovery period, the degree of weight loss in the male 200 mg/kg/day and female 60 mg/kg/day groups slowed (at week 17, males decreased by 6.8% and females decreased by 8.2%). Although there was some recovery of weight lost, it is considered a toxicological effect because the weight loss was > 10% of the control group, and a dose correlation was observed (Fig. 3).

#### Food consumption

**Fig. 4:**
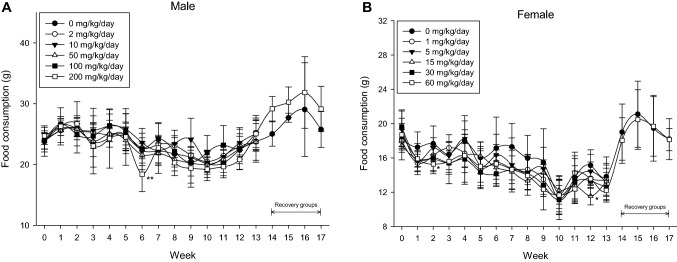
Food consumption of rats in 90-day repeated oral toxicity study. Food consumption of male (**A**) and female (**B**) rats in 13-week repeated oral dose administration of BPF (*n* = 10 per each groups). Error bar represents standard deviation. **Significant changes when compared to its vehicle control group, *p* < 0.01, *significant changes when compared to its vehicle control group, *p* < 0.05

In the male 200 mg/kg/day group and the female 15 and 60 mg/kg/day groups, sporadic decreases in feed intake were observed, but there was no toxicological significance as it was a temporary change (Fig. 4).

#### Ophthalmological examination

No abnormal findings were observed in all test animals subjected to ophthalmological examinations in the main and the recovery groups.

#### Clinical pathology examination

- *Urinalysis and hematology:* No changes related to the test substance were found.
- *Blood coagulation test:* In the main group, the prothrombin time (PT) level of the male 200 mg/kg/day and the females dosed > 15 mg/kg/day was higher than that of the control group. The response showed a dose dependency and, in particular, both sexes showed the same tendency in the highest dose group. The increase in PT values at the highest dosages exceeded the range of biological fluctuations (♂ 9.01–11.15 s, ♀ 7.90–10.06 s) at this laboratory. The changes in PT were considered to be an effect of the test substance for two reasons: (1) fat-soluble vitamins are required for the production of coagulation factors related to PT levels. Among the histopathological findings of this study, lymphatic dilatation seems to have inhibited the absorption of fat-soluble nutrients, and (2) the PT level recovered to some extent during the recovery period without administration of the substance. The increase in APTT levels in the recovery group of females receiving 60 mg/kg/day group was not observed in the main group, so it seems that this is of no toxicological significance (Table 4)
- *Blood biochemistry:* In the main group, total cholesterol (CHO), high-density lipoproteins (HDL), and low-density lipoproteins levels (LDL) in the males dosed > 50 mg/kg/day and the CHO level in the females dosed > 5 mg/kg/day groups decreased. In the case of HDL, a decreasing trend was observed. It was judged a secondary effect of absorption inhibition of the test substance (refer to histopathological examination results.).
  The cholinesterase (ChE) levels decreased in the male 200 mg/kg/day group in the main group and the recovery group, and there was no statistical difference, but a decreasing trend was observed even at high dosages in the female group, which is considered to be a change by the test substance. However, both male and female levels were within the biological rage of the test animals (♂ 101–325 U/l, ♀ 909–2449 U/l), therefore, its toxicological significance is unclear (Table 5).
- *Hormone analysis:* In the main group, the male 200 mg/kg/day thyroxine (T4) level increased, therefore, an effect of the test substance cannot be excluded. The relative weight of the right thyroid gland of the male 200 mg/kg/day group and the left thyroid gland of the female 30 mg/kg/day group was increased compared to that of the control. The incidence was unilateral, there was no difference in absolute weight, and the dose dependency was not clear, so it was considered to be a relative change due to weight loss of the test animal (about 8–17% decrease in fasting weight before postmortem compared to the control group). In the case of histopathological examination, no functional or morphological abnormalities of the thyroid gland were identified and recovery was demonstrated during the recovery period. Therefore, the change in the T4 level was not found to have any supporting results, therefore, it was judged that there was no toxicological significance in the hormone analysis (Table 6).

**Table 3:** Pharmacokinetics parameters of BPF

		Male		Female	
AUC_0-72 h_	(ng·h/ml)	15112.95 ± 4854.7	(5)	23377.32 ± 4478.28	(5)
C_max_	(ng/ml)	12552.67 ± 3468.59	(5)	16101.55 ± 4016.43	(5)
T_max_	(h)	0.25 ± 0.00	(5)	0.25 ± 0.00	(5)
Vd	(ng/kg)	194639.26 ± 101617.42	(5)	216032.65 ± 76471.91	(5)
T_1/2_	(h)	9.02 ± 2.69	(5)	21.77 ± 3.96	(5)
Cl	(ml/h/kg)	14642.2 ± 5763.66	(5)	9581.73 ± 1294.14	(5)

*AUC* Area under the curve, *C*_*max*_ the peak plasma concentration of a drug after administration, *T*_*max*_ time to reach C_max_, *Vd* volume of distribution, *T*_*1/2*_ absorption half-life, *Cl* clearance

200 mg/ml of BPF was treated in male and female SD rats. *N* = 5

**Table 4:** Summary of coagulation test in 90-day repeated oral dose of BPF

		GROUP (mg/kg/day)											
Sex: male		G1 (0)		G2 (2)		G3 (10)		G4 (50)		G5 (100)		G6 (200)	
Main group	PT^1^	10.41 ± 0.55	(10)	10.65 ± 0.75	(10)	10.41 ± 0.44	(10)	11.03 ± 0.63	(10)	10.97 ± 0.66	(10)	12.16** ± 0.73	(10)
	APTT^2^	15.4 ± 1.7	(10)	15.4 ± 1.4	(10)	15.6 ± 1.4	(10)	15.6 ± 1.5	(10)	15.3 ± 1.2	(10)	15.9 ± 1.0	(10)
Recovery group	PT	10.85 ± 1.43	(5)									10.40 ± 0.14	(5)
	APTT	17.0 ± 1.1	(5)									17.3 ± 0.9	(5)

Sex: female		G1 (0)		G2 (1)		G3 (5)		G4 (15)		G5 (30)		G6 (60)	
Main group	PT	8.88 ± 0.68	(10)	8.94 ± 0.56	(10)	9.09 ± 0.41	(10)	9.49** ± 0.56	(10)	9.79** ± 0.53	(10)	10.23** ± 0.59	(10)
	APTT	15.8 ± 1.5	(10)	15.9 ± 1.3	(10)	15.4 ± 1.4	(10)	16.4 ± 1.5	(10)	15.8 ± 1.9	(10)	15.9 ± 1.0	(10)
Recovery group	PT	9.21 ± 0.44	(5)									9.21 ± 0.36	(5)
	APTT	17.1 ± 0.9	(5)									18.6* ± 0.4	(5)

Mean ± S.D (number of animals). 1: Prothrombin time, 2: active partial thromboplastin time

**Significant difference compared with control group value, *p* < 0.01

**Table 5:** Summary of serum biochemical test in 90-day repeated oral dose of BPF

		Group (mg/kg/day)																							
Sex: male		G1 (0)				G2 (2)				G3 (10)				G4 (50)				G5 (100)				G6 (200)			
Main group	CHO^1^ (mg/dl)	77	±	15	(10)	67	±	13	(10)	66	±	10	(10)	54**	±	12	(10)	50**	±	11	(10)	35**	±	9	(10)
	HDL^2^ (mg/dl)	22	±	3	(10)	20	±	3	(10)	20	±	2	(10)	17**	±	3	(10)	16**	±	3	(10)	12**	±	3	(10)
	LDL^3^ (mg/dl)	7	±	2	(10)	7	±	2	(10)	6	±	1	(10)	5**	±	1	(10)	4**	±	1	(10)	3**	±	1	(10)
	ChE^4^ (U/l)	247	±	39	(10)	246	±	46	(10)	257	±	47	(10)	222	±	78	(10)	204	±	52	(10)	161**	±	29	(10)
Recovery group	CHO (mg/dl)	74	±	31	(5)																	66	±	9	(5)
	HDL (mg/dl)	18	±	5	(5)																	18	±	2	(5)
	LDL (mg/dl)	8	±	3	(5)																	6	±	2	(5)
	ChE (U/l)	166	±	63	(5)																	92*	±	19	(5)

Sex: female		G1 (0)				G2 (1)				G3 (5)				G4 (15)				G5 (30)				G6 (60)			
Main group	CHO (mg/dl)	85	±	15	(10)	84	±	12	(10)	73**	±	9	(10)	60**	±	8	(10)	59**	±	11	(10)	49**	±	14	(10)
	HDL (mg/dl)	26	±	3	(10)	26	±	3	(10)	24	±	2	(10)	21*	±	3	(10)	21	±	3	(10)	18^*^	±	6	(10)
	LDL (mg/dl)	5	±	1	(10)	6	±	1	(10)	5	±	1	(10)	4**	±	1	(10)	4	±	1	(10)	5	±	1	(10)
	ChE(U/l)	2058	±	570	(10)	2272	±	501	(10)	2235	±	527	(10)	1800	±	484	(10)	1798	±	318	(10)	1676	±	266	(10)
Recovery group	CHO (mg/dl)	108	±	28	(5)																	111	±	16	(5)
	HDL (mg/dl)	28	±	6	(5)																	30	±	3	(5)
	LDL (mg/dl)	7	±	2	(5)																	5	±	1	(5)
	ChE (U/l)	990	±	286	(5)																	947	±	269	(5)

Mean ± S.D (number of animals). 1: Total cholesterol, 2: high-density lipoproteins, 3: low-density lipoproteins, 4: cholinesterase

**Significant difference compared with control group value, *p* < 0.01, *Significant difference compared with control group value, *p* < 0.05

*Organ weights:* As a result of organ weight measurement, no toxicological changes related to the test substance were observed.

#### Estrus cycle examination

There was no statistically significant difference observed between the test and recovery groups in the female rats (Table 7).

**Table 6:** Summary of hormone analysis in 90-day repeated oral dose of BPF

			GROUP (mg/kg/day)																							
Sex: male			G1 (0)				G2 (2)				G3 (10)				G4 (50)				G5 (100)				G6(200)			
Main group		T4^1^ (ng/ml)	52.57	±	2.40	(10)	51.87	±	1.99	(10)	51.77	±	3.89	(10)	53.58	±	2.74	(10)	52.49	±	3.27	(10)	57.15^**^	±	5.06	(10)
		T3^2^ (ng/ml)	0.736	±	0.129	(10)	0.829	±	0.163	(10)	0.746	±	0.190	(10)	0.763	±	0.137	(10)	0.802	±	0.103	(10)	0.874	±	0.225	(10)
		TSH^3^ (ng/ml)	2.495	±	0.557	(10)	2.091	±	0.698	(10)	2.076	±	0.784	(10)	1.963	±	0.474	(10)	2.118	±	0.674	(10)	2.301	±	0.534	(10)
Recovery group		T4 (ng/ml)	48.83	±	3.62	(5)																	49.69	±	4.36	(5)
		T3 (ng/ml)	0.689	±	0.108	(5)																	0.547	±	0.093	(5)
		TSH (ng/ml)	4.287	±	1.107	(5)																	4.644	±	2.247	(5)

Sex: female			G1 (0)				G2 (1)				G3 (5)				G4 (15)				G5 (30)				G6(60)			
Main group		T4 (ng/ml)	51.70	±	4.87	(10)	51.39	±	7.19	(10)	49.09	±	6.27	(10)	48.84	±	5.97	(10)	55.42	±	5.12	(10)	55.34	±	4.13	(10)
		T3 (ng/ml)	0.845	±	0.199	(10)	0.762	±	0.212	(10)	0.861	±	0.246	(10)	0.858	±	0.238	(10)	0.952	±	0.194	(10)	0.941	±	0.295	(10)
		TSH (ng/ml)	1.448	±	0.442	(10)	1.224	±	0.357	(10)	1.220	±	0.233	(10)	1.602	±	0.556	(10)	1.453	±	0.454	(10)	1.663	±	0.783	(10)
		E2^4^ (pg/ml)	374.54	±	100.62	(10)	428.99	±	132.73	(10)	429.41	±	142.26	(10)	440.86	±	137.48	(10)	415.98	±	162.53	(10)	376.35	±	139.63	(10)
Recovery group		T4 (ng/ml)	45.89	±	5.36	(5)																	44.85	±	3.75	(5)
		T3 (ng/ml)	0.574	±	0.154	(5)																	0.588	±	0.043	(5)
		TSH (ng/ml)	2.420	±	0.836	(5)																	2.554	±	0.903	(5)
		E2 (pg/ml)	237.46	±	27.23	(5)																	244.75	±	41.25	(5)

Mean ± S.D (number of animals)

1: Thyroxine, 2: triiodothyronine, 3: thyroid-stimulating hormone, 4: oestradiol

**Significant difference compared with control group value, *p* < 0.01

#### Necropsy and histopathological examination

**Fig. 5:**
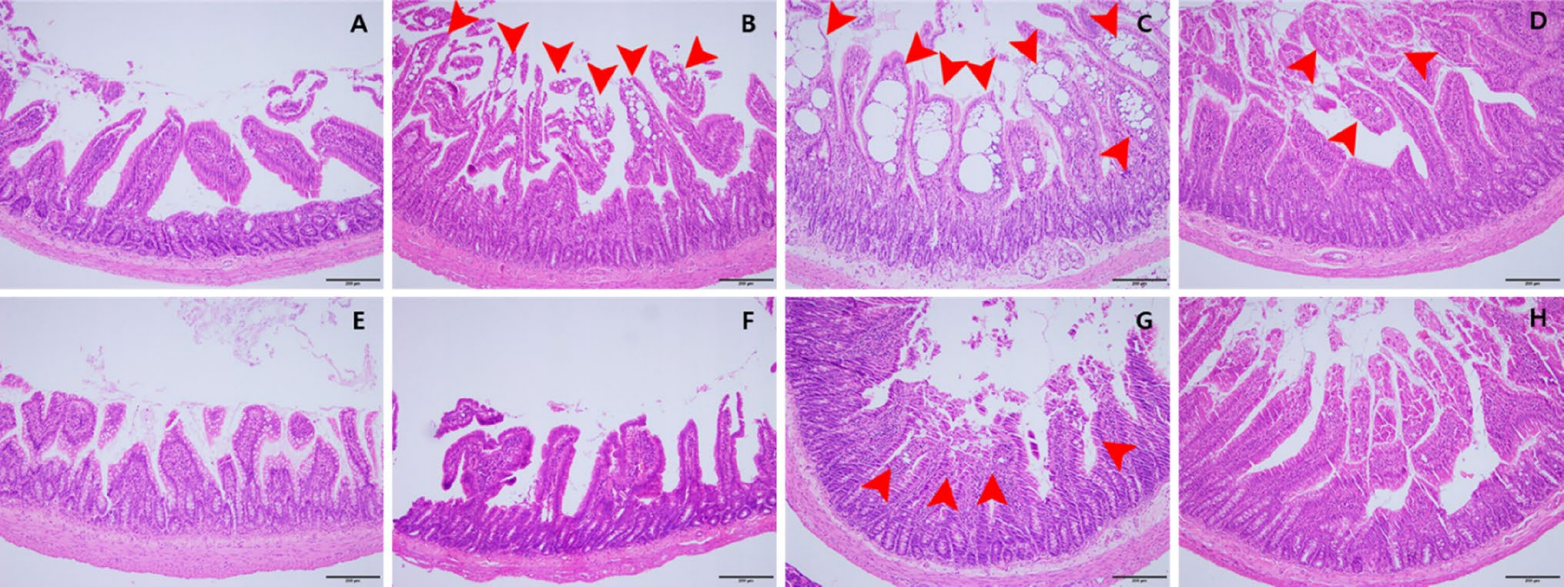
Lacteal dilatation in small intestine after 90-day repeated oral dose of BPF. **A** Male control group, normal duodenum, **B** male 100 mg/kg/day group, duodenum with mild dilated lacteals, **C** male 200 mg/kg/day group, duodenum with severely dilated lacteals, **D** male recovery group, duodenum with minimally dilated lacteals, **E** female control group, normal duodenum, **F** female 30 mg/kg/day group, normal duodenum, **G** female 60 mg/kg/day group, duodenum with minimally dilated lacteals, **H** female recovery group, normal duodenum (H&E staining, X100, scale bar represents 200 um)

As a result of the necropsy, there were no gross abnormal findings related to the BPF administration. At microscopic findings, in main group, males dosed > 10 mg/kg/day groups and females dosed 15 mg/kg/day and 60 mg/kg/day groups showed minimal to severe lacteal dilatation in the small intestine (duodenum or ileum). In the recovery group, a minimal level of lymphatic dilatation was observed in the male 200 mg/kg/day and female 60 mg/kg/day groups, and symptoms recovered (Fig. 5 and Table 8).

**Table 7:** Summary of estrus cycle examination in 90-day repeated oral dose of BPF

	GROUP (mg/kg/day)												
Sex: female		G1 (0)		G2 (1)		G3 (5)		G4 (15)		G5 (30)		G6 (60)	
		*N*	%	*N*	%	*N*	%	*N*	%	*N*	%	*N*	%
Main	Proestrus	0/10	0	1/10	10	1/10	10	0/10	0	0/10	0	0/10	0
	Estrus	2/10	20	4/10	40	3/10	30	2/10	20	4/10	40	4/10	40
	Metestrus	2/10	20	0/10	0	0/10	0	2/10	20	0/10	0	1/10	10
	Diestrus	6/10	60	5/10	50	6/10	60	6/10	60	6/10	60	5/10	50
Recovery	Proestrus	0/5	0									0/5	0
	Estrus	2/5	40									1/5	20
	Metestrus	0/5	0									0/5	0
	Diestrus	3/5	60									4/5	80

Number of animals with the sign/number of animals examined

**Table 8:** Summary of histological findings (lacteal dilatation) in 90-day repeated oral dose of BPF

	Organs	Signs		Group (mg/kg/day)											
Sex: male				G1 (0)		G2 (2)		G3 (10)		G4 (50)		G5 (100)		G6 (200)	
				*N*	%	*N*	%	*N*	%	*N*	%	*N*	%	*N*	%
Main group	Duodenum	No remarkable lesions		10/10	100	10/10	100	9/10	90	7/10	70	1/10	10	1/10	10
		Remarkable lesions		0/10	0	0/10	0	1/10	10	3/10	30	9/10	90	9/10	90
		Lacteal dilatation	±	0/10	0	0/10	0	1/10	10	3/10	30	6/10	60	2/10	20
			+	0/10	0	0/10	0	0/10	0	0/10	0	3/10	30	2/10	20
			+ +	0/10	0	0/10	0	0/10	0	0/10	0	0/10	0	3/10	30
			+ + +	0/10	0	0/10	0	0/10	0	0/10	0	0/10	0	2/10	20
Recovery group	Duodenum	No remarkable lesions		5/5	100									1/5	20
		Remarkable lesions		0/5	0									4/5	80
		Lacteal dilatation	±	0/5	0									4/5	80

Sex: female				G1 (0)		G2 (1)		G3 (5)		G4 (15)		G5 (30)		G6 (60)	
				N	%	N	%	N	%	N	%	N	%	N	%
Main group	Duodenum	No remarkable lesions		10/10	100	10/10	100	10/10	100	10/10	100	10/10	100	7/10	70
		Remarkable lesions		0/10	0	0/10	0	0/10	0	0/10	0	0/10	0	3/10	30
		Lacteal dilatation	±	0/10	0	0/10	0	0/10	0	0/10	0	0/10	0	3/10	30
	Ileum	No remarkable lesions		10/10	100	10/10	100	10/10	100	9/10	90	10/10	100	9/10	90
		Remarkable lesions		0/10	0	0/10	0	0/10	0	1/10	10	0/10	0	1/10	10
		Lacteal dilatation	±	0/10	0	0/10	0	0/10	0	1/10	10	0/10	0	1/10	10
Recovery group	Duodenum	No remarkable lesions		5/5	100									4/5	80
		Remarkable lesions		0/5	0									1/5	20
		Lacteal dilatation	±	0/5	0									1/5	20

#### NOAEL (the-no-observed-adverse-effect level) and NOEL (no-observed-effect level)

**Fig. 6:**
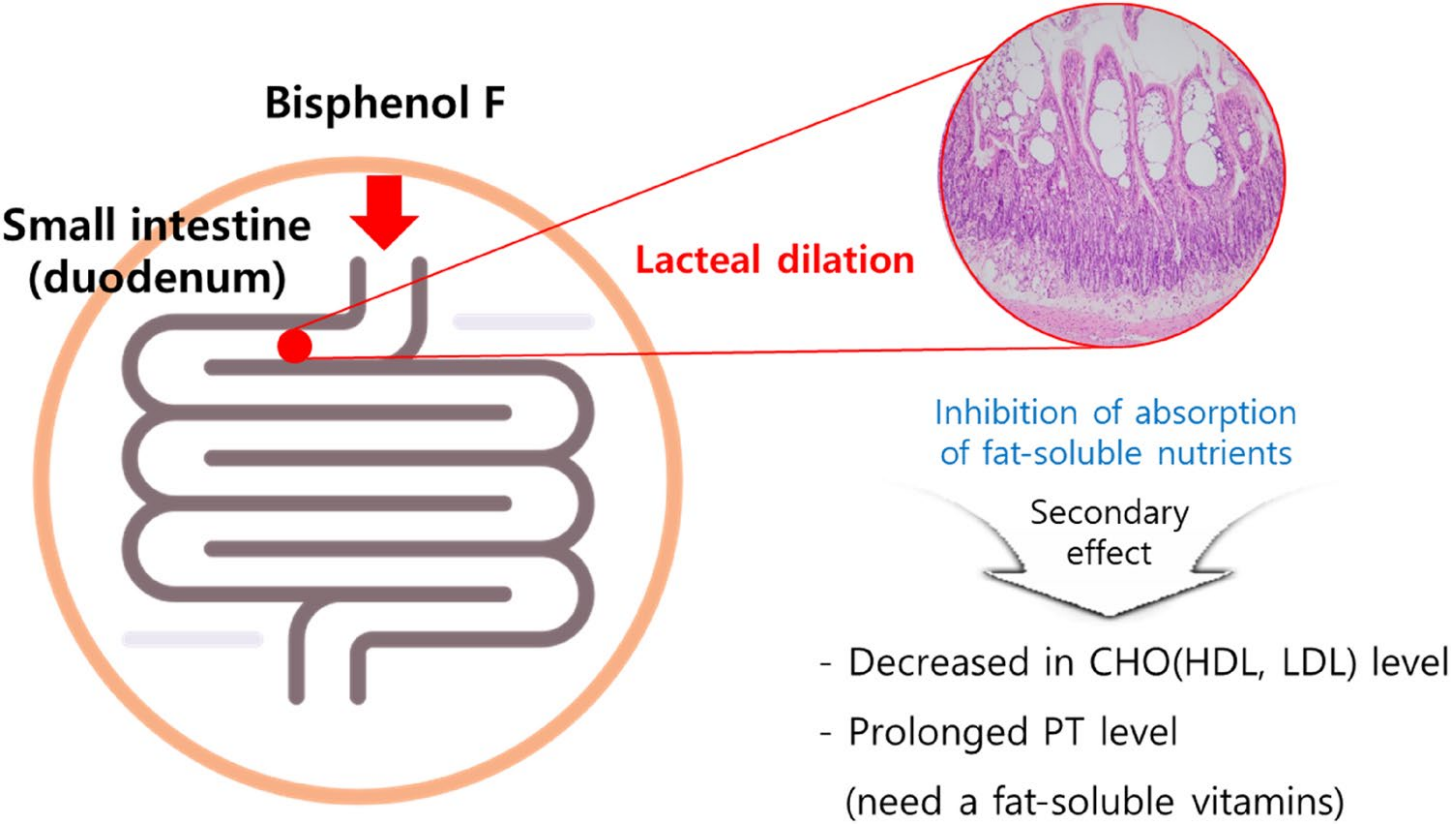
Summary of mechanism of BPF in rat after 90-day repeated oral administration

Under the test conditions, weight loss was observed in the males dosed > 200 mg/kg/day and females dosed > 30 mg/kg/day. Lacteal dilatation in the small intestine was observed in the males dosed > 10 mg/kg/day and females dosed > 15 mg/kg/day. Secondary to lacteal dilatation, the CHO level decreased in the males dosed > 50 mg/kg/day and the females dosed over > 5 mg/kg/day. The PT value was prolonged in the male 200 mg/kg/day and female > 15 mg/kg/day groups. All the findings mentioned above showed reversibility during the recovery period in the absence of BPF (Fig. 6). Considering of all the test result, the NOAEL of BPF was determined as 2 mg/kg/day in males and 5 mg/kg/day in females, and the NOEL of BPF was determined as 2 mg/kg/day in males and 1 mg/kg/day in females. The target organ was identified as the small intestine.

Therefore, the NOAEL of BPF was determined as 2 mg/kg/day in males and 1 mg/kg/day in females, and the target organ was identified as the small intestine.

### Genotoxicity study

#### Reverse mutation test

As a result of the main test, growth inhibition of bacteria was observed in all strains in the direct and metabolic activity methods. Compared to the negative control group, no increase in the number of reverse mutated colonies (which could be judged as a positive result) was observed. As a result of the sterility confirmation test of the test substance and S9 mix, no contamination by microorganisms was observed in the positive and negative controls. The number of colonies induced was within the expected range, indicating that each test was performed properly. From these results, it was determined that BPF did not induce a reverse mutation under these test conditions.

#### Chromosomal abnormality test

In the case of the 24-h continuous treatment group and the 18-h recovery after 6-h treatment group without metabolic activity, the frequency of abnormal intermediate phases increased significantly compared to the control group at all dosages. In the case of the metabolic activity method (6-h treatment and 18-h recovery), no statistically significant increase was observed in the frequency of abnormal metaphase in all treatment groups compared to the negative control group. In the direct method and the metabolic activity method, the frequency of ploidy and intranuclear embedding also failed to increase compared to the negative control. Consequently, BPF did not induce chromosomal abnormalities in CHO-k1 cells under these test conditions.

#### In vivo micronucleus test

As a result of the main test, no dead animals occurred in the test substance administration groups, and abnormal walking was seen in animals receiving 2000 mg/kg/day group. The number of polyinflammatory red blood cells among the total red blood cells did not show a distinct difference, and no inhibition of the proliferation of myeloid cells was observed compared to the control group. The micronucleus induction frequency observed in 4000 polyinflammatory red blood cells per animal did not show statistically significant results compared to the control at all dosages. Meanwhile, a difference was observed in the micronucleus induction frequency of the positive control group compared to the excipient control group (*p* < 0.01). After administration of the test substance, no statistically significant body weight change was observed in all administration groups compared to the control. The micronucleus incidence in the control and the positive control and the proportion of polyinflammatory red blood cells among the total red blood cells were within the range of historical control data. From the above results, it is believed that BPF does not induce micronuclei in the bone marrow cells of mice under the conditions of this test.

## Discussion

BPF, which is used as a substitute for BPA, is a household chemical that cannot be avoided because it is widely prevalent in daily life but concerns about its safety have recently increased (Song et al. 2017). Unlike BPA, where many studies on safety have been conducted, access and acquisition of information on BPF is very limited (Moon 2019). Therefore, through this study, we attempted to produce accurate and reliable information on toxicity results, kinetics, and exposure levels, and to use them as basic data for various derived pathways in social, policy, and scientific fields.

In the pharmacokinetic study, it was confirmed that single oral exposure to BPF resulted in systemic exposure, and a pharmacokinetic profile was obtained. BPF has a high elimination half-life, therefore, drug release was delayed. In addition, females showed a higher absorption rate and an increase in elimination half-life, resulting in a lower excretion rate, which further delayed drug loss compared to that in males.

When the microbial reverse mutation test and the chromosomal aberration test using CHO-k1 cells (genotoxicity tests to determine carcinogenicity) were performed, BPF did not induce reversion mutation or chromosomal aberration. In the in vivo micronucleus test, it was confirmed that BPF did not induce micronuclei in the bone marrow cells of mice. The above three genotoxicity tests confirmed that BPF does not induce genotoxicity.

As a result of the single oral dose toxicity test for BPF, symptoms such as confusion and convulsions were observed when administered orally in a single-dose toxicity test in rodents, and the approximate lethal dose was found to be 4000 mg/kg. As a result of a repeated oral administration toxicity test over 90-day, weight loss in the male 200 mg/kg/day and female > 30 mg/kg/day groups, lacteal dilatation in small intestine in the male > 10 mg/kg/day and female > 15 mg/kg/day groups, reduction of total cholesterol in males > 50 mg/kg/day and females > 5 mg/kg/day, and prolonged prothrombin time in males 200 mg/kg/day and females > 15 mg/kg/day were observed. All the findings mentioned above showed reversibility during the recovery period in the absence of BPF. Through the above test results, the NOAEL of BPF was determined as 2 mg/kg/day in males and 5 mg/kg/day in females, and the NOEL of BPF was determined as 2 mg/kg/day in males and 1 mg/kg/day in females. The target organ was identified as the small intestine.

**Fig. 7:**
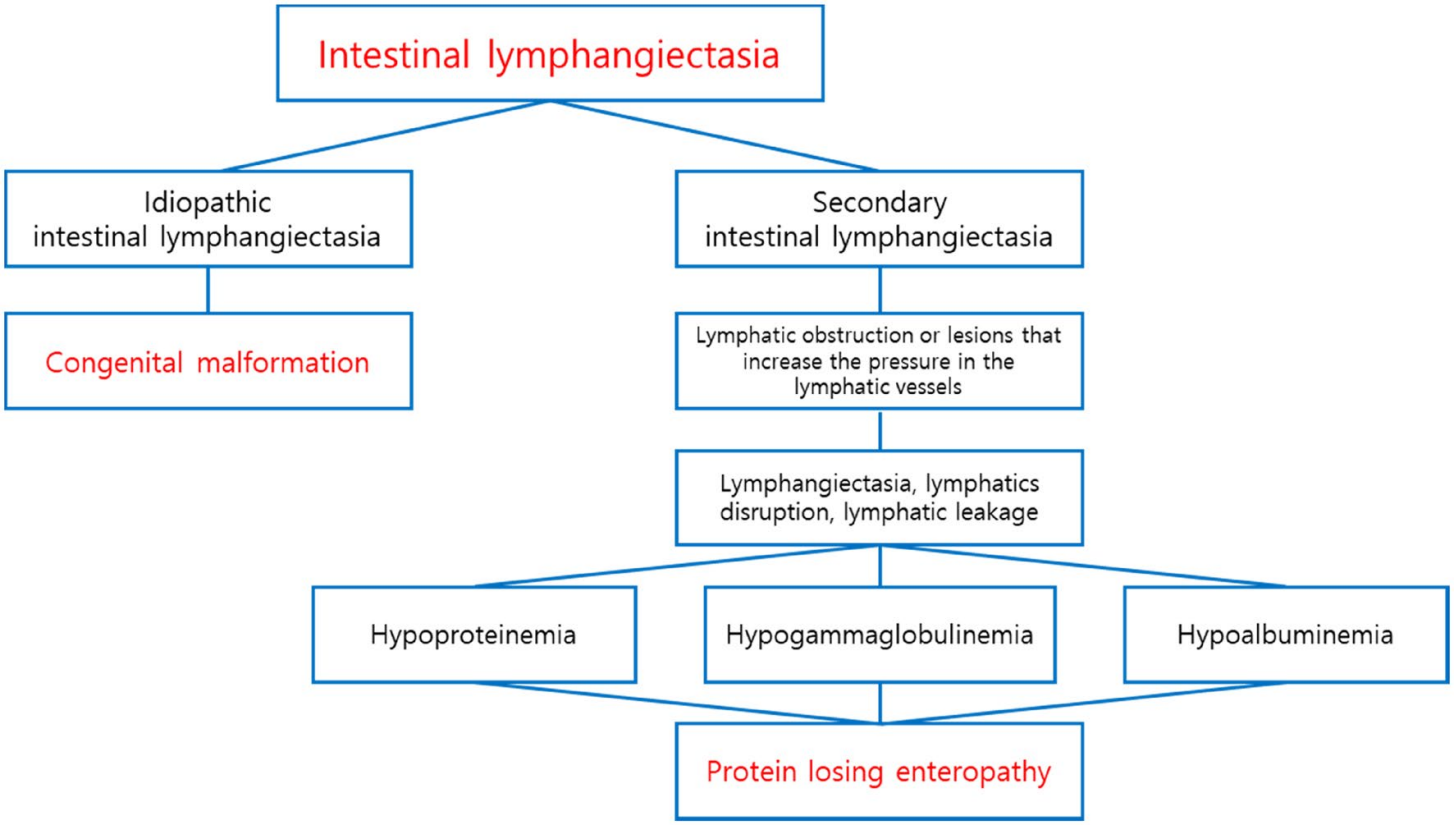
Types of intestinal lymphangiectasia observed in humans and diagnosis

Lacteal dilatation in the small intestine may be associated with intestinal lymphangiectasia in humans. Intestinal lymphangiectasis is classified into idiopathic intestinal lymphangiectasia due to congenital malformation and secondary intestinal lymphangiectasia caused by lymphatic obstruction or an underlying disease that increases intra-lymphatic pressure (Vignes and Bellanger 2008; Wen et al. 2010). When the pressure of the lymphatic vessels increases, the lymphatic vessels may expand and rupture, and the lymphatic fluid may leak, resulting in protein-losing enteropathy accompanied by hypoproteinemia, hypoalbuminemia, and hypogammaglobulinemia (Umar and Dibaise 2010). In the postmortem findings, clinical pathology, and organ weight results, no changes suggestive of protein loss or intestinal disease were observed, and there was no cellular damage, degenerative change, or inflammation, and the lesion occurred in the small intestine (especially in the duodenum), not the entire intestine, which is considered to be a local effect that occurred during absorption of BPF (Fig. 7). However, lacteal dilatation is not commonly observed in rats (Boyle et al. 2012), and the incidence and severity of lesions were dose dependent. In addition, it is judged to be the effect of BPF on the function of the lymphatic vessels to absorb fat-soluble nutrients, the prolongation of the PT affected by fat-soluble vitamin-related coagulation factors, and the decrease in the blood CHO level. As a result of the histopathological examination, lateral dilatation showed reversibility at a highest dose. In addition, no protein loss observed as would be expected for lymphangiectasia, therefore, the observed dilatation seems to be an adaptive effect.

Several studies have reported estimated daily intakes through exposure scenarios of BPA including its analogs (Liao and Kannan 2013; Hartle et al. 2016; Wang et al. 2020). The above reports may suggest that the NOAEL we determined also deviated somewhat from the actual exposure level. However, since the amount of daily exposure to BPF varies from region to region, accurate scenario studies have not been conducted, and risk assessments must consider the fact that people may be unintentionally exposed to BPF throughout their lives, it is recommended to set high safety factor limits. In terms of aspects, the NOAEL and NOEL setting in this test was meaningful.

In the recent research, BPF was detected in several mustard products containing S. alba seeds and in various plants of the orchid family (Zoller et al. 2015). BPF in mustard and other plant species is not derived from synthetic materials, packaging, or other contaminants, but is most likely formed as a breakdown product of glucosinolates (Lietzow 2021). Whether mustard is the only natural cause of BPF, or whether human exposure also stems from other natural constituents of human food, may be an issue. However, from a toxicological point of view, the exposure to BPF by intake of mustard is thought to be very negligible. The reason is that, as a food additive, human cannot consume an excessive amount under normal circumstances, and individual can control the intake of BPF by selectively consuming mustard as a preference food. Except for the intentional intake of mustard, BPF as a substitute for BPA can be unintentionally long-term exposure to an unspecified number of people, so it is necessary to control the exposure amount of BPF. Recently, due to a variety of incidents, accidents involving chemicals have increased the aversion and concerns about chemical usage (Kwon et al. 2020). Reliable toxic information can relieve public anxiety about BPF and can increase public health safety through scientific safety management by the relevant authorities (Vogel 2012). This can have concomitant effects on the usage of household products, and downstream manufacturing, transportation, and retail.

Currently, BPF is being used without a suitable substitute, but a risk issue has been raised which mandates national policy intervention for its use (Eladak et al. 2015). As for the ideal safety management level of products that are directly applied to the human body, strict standards that do not harm the health of the people should be given top priority. But, at the same time the boundary should not regulate the industry more than necessary. This requires a full safety assessment of BPF. The toxicity test data produced in this study can be used as a scientific basis for safety management and can be used as basic data for risk assessment and policy establishment regarding the use of and exposure to BPF in the future. Through press releases and educational materials such as the correct selection/use/disposal of chemical products containing BPF, efforts can be made to improve public health and quality of life worldwide.

## Abbreviations

AUC: Area under the curve
BPA: Bisphenol A
BPF: Bisphenol F
BW: Body weight
Cl: Clearance
C_max_: Peak plasma concentration of a drug after administration
LC/MS/MS: Liquid chromatography with tandem mass spectrometry
NCA: Necessary condition analysis
NOAEL: No-observed-adverse-effect level
NOEL: No-observed-effect level
SD: Sprague–Dawley
T1/2: Absorption half-life
T_max_: Time to reach C_max_
U.S. FDA: United States Food and Drug Administration
Vd: Volume of distribution

## References

[CR1] Andra SS, Charisiadis P, Arora M (2015). Biomonitoring of human exposures to chlorinated derivatives and structural analogs of bisphenol A. Environ Int.

[CR2] Bae S, Kim JH, Lim YH (2012). Associations of bisphenol a exposure with heart rate variability and blood pressure. Hypertension.

[CR3] Board of Scientific Counselors (2008) National toxicology program, 2008. In: National toxicology program

[CR4] Boyle MC, Crabbs TA, Wyde ME (2012). Intestinal lymphangiectasis and lipidosis in rats following subchronic exposure to indole-3-carbinol via oral gavage. Toxicol Pathol.

[CR5] Cabaton N, Chagnon MC, Lhuguenot JC (2006). Disposition and metabolic profiling of bisphenol F in pregnant and nonpregnant rats. J Agric Food Chem.

[CR6] Chen D, Kannan K, Tan H (2016). Bisphenol analogues other than BPA: environmental occurrence, human exposure, and toxicity—a review. Environ Sci Technol.

[CR7] Eladak S, Grisin T, Moison D (2015). A new chapter in the bisphenol a story: bisphenol S and bisphenol F are not safe alternatives to this compound. Fertil Steril.

[CR8] Goldinger DM, Demierre AL, Zoller O (2015). Endocrine activity of alternatives to BPA found in thermal paper in Switzerland. Regul Toxicol Pharmacol.

[CR9] Hartle JC, Navas-Acien A, Lawrence RS (2016). The consumption of canned food and beverages and urinary Bisphenol A concentrations in NHANES 2003–2008. Environ Res.

[CR10] Heimeier RA, Shi YB (2010). Amphibian metamorphosis as a model for studying endocrine disruption on vertebrate development: effect of bisphenol A on thyroid hormone action. Gen Comp Endocrinol.

[CR11] Higashihara N, Shiraishi K, Miyata K (2007). Subacute oral toxicity study of bisphenol F based on the draft protocol for the “Enhanced OECD Test Guideline no. 407”. Arch Toxicol.

[CR12] Hormann AM, Vom Saal FS, Nagel SC (2014). Holding thermal receipt paper and eating food after using hand sanitizer results in high serum bioactive and urine total levels of bisphenol A (BPA). PLoS ONE.

[CR13] Huang GM, Tian XF, Fang XD, Ji FJ (2016). Waterborne exposure to bisphenol F causes thyroid endocrine disruption in zebrafish larvae. Chemosphere.

[CR14] Ijaz S, Ullah A, Shaheen G, Jahan S (2020). Exposure of BPA and its alternatives like BPB, BPF, and BPS impair subsequent reproductive potentials in adult female Sprague Dawley rats.

[CR15] Joe M, Braun RH (2011). Bisphenol A and children’s health. Curr Opin Pediatr.

[CR16] Kang JH, Kondo F, Katayama Y (2006). Human exposure to bisphenol A. Toxicology.

[CR17] Kwon SA, Yoo HJ, Song E (2020). Korean consumers’ recognition of risks depending on the provision of safety information for chemical products. Int J Environ Res Public Health.

[CR18] Liao C, Kannan K (2013). Concentrations and profiles of bisphenol A and other bisphenol analogues in foodstuffs from the United States and their implications for human exposure. J Agric Food Chem.

[CR19] Lietzow J (2021). Biologically active compounds in mustard seeds: a toxicological perspective. Foods.

[CR20] Maffini MV, Rubin BS, Sonnenschein C, Soto AM (2006). Endocrine disruptors and reproductive health: the case of bisphenol-A. Mol Cell Endocrinol.

[CR21] Mikołajewska K, Stragierowicz J, Gromadzińska J (2015). Bisphenol A—application, sources of exposure and potential risks in infants, children and pregnant women. Int J Occup Med Environ Health.

[CR22] Moon MK (2019). Concern about the safety of bisphenol a substitutes. Diabetes Metab J.

[CR23] Moriyama K, Tagami T, Akamizu T (2002). Thyroid hormone action is disrupted by bisphenol A as an antagonist. J Clin Endocrinol Metab.

[CR24] Nah WH, Park MJ, Gye MC (2011). Effects of early prepubertal exposure to bisphenol A on the onset of puberty, ovarian weights, and estrous cycle in female mice. Clin Exp Reprod Med.

[CR25] Rochester JR (2013). Bisphenol A and human health: A review of the literature. Reprod Toxicol.

[CR26] Rochester JR, Bolden AL (2015). Systematic review and comparison of the hormonal activity. Environ Health Perspect.

[CR27] Ruan T, Liang D, Song S (2015). Evaluation of the in vitro estrogenicity of emerging bisphenol analogs and their respective estrogenic contributions in municipal sewage sludge in China. Chemosphere.

[CR28] Song CY, Kim W, Gye MC (2017). Current state of use and the risks of bisphenols: A minireview. Environ Biol Res.

[CR29] Staples CA, Dorn PB, Klecka GM (1998). A review of the environmental fate, effects, and exposures of bisphenol A. Chemosphere.

[CR30] Tewar S, Auinger P, Braun JM (2016). Association of bisphenol A exposure and attention-deficit/hyperactivity disorder in a national sample of U.S. children. Environ Res.

[CR31] Tsai WT (2006). Human health risk on environmental exposure to bisphenol-A: a review. J Environ Sci Heal Part C Environ Carcinog Ecotoxicol Rev.

[CR32] Ullah A, Pirzada M, Jahan S (2019). Prenatal BPA and its analogs BPB, BPF, and BPS exposure and reproductive axis function in the male offspring of Sprague Dawley rats. Hum Exp Toxicol.

[CR33] Umar SB, Dibaise JK (2010). Protein-losing enteropathy: case illustrations and clinical review. Am J Gastroenterol.

[CR34] Usman A, Ikhlas S, Ahmad M (2019). Occurrence, toxicity and endocrine disrupting potential of bisphenol-B and bisphenol-F: a mini-review. Toxicol Lett.

[CR35] Vandenberg LN, Hauser R, Marcus M (2007). Human exposure to bisphenol A (BPA). Reprod Toxicol.

[CR36] Vignes S, Bellanger J (2008). Primary intestinal lymphangiectasia (Waldmann’s disease). Orphanet J Rare Dis.

[CR37] Vogel SA (2012). Is it safe?: BPA and the struggle to define the safety of chemicals.

[CR38] Wang H, Liu Z, hua, Tang Z, (2020). Bisphenol analogues in Chinese bottled water: quantification and potential risk analysis. Sci Total Environ.

[CR39] Wen J, Tang Q, Wu J (2010). Primary intestinal lymphangiectasia: four case reports and a review of the literature. Dig Dis Sci.

[CR40] Yamazaki E, Yamashita N, Taniyasu S (2015). Bisphenol A and other bisphenol analogues including BPS and BPF in surface water samples from Japan, China, Korea and India. Ecotoxicol Environ Saf.

[CR41] Yong Eui K, MyungSil H, You-Gyoung P et al (2018) Study on the exposure assessment system based on human biomonitoring(I) (Total exposure assessment of bisphenols, acrylamides, furans)

[CR42] Yoo J, Baek Y, Jeon H et al (2016) Korean National Environmental Health Survey (KoNEHS)—Annual Report on Third stage , 2 nd year (2016)—Environmental Health Research Department. Environmental Health Research Division Environmental Health Research Department National Institute of Environmental Research

[CR43] Zoller O, Brüschweiler BJ, Magnin R (2015). Natural occurrence of bisphenol F in mustard. Food Addit Contam Part A Chem Anal Control Expo Risk Assess.

